# Lathyrol Exerts Anti-Pulmonary Fibrosis Effects by Activating PPARγ to Inhibit the TGF-β/Smad Pathway

**DOI:** 10.3390/ijms27010387

**Published:** 2025-12-30

**Authors:** Qian Zeng, Min-Lin Liao, Yu-Yang Luo, Shuang Li, Gao You, Chong-Mei Huang, Min-Hui Liu, Wei Liu, Si-Yuan Tang

**Affiliations:** 1 Xiangya Nursing School, Central South University, 172 Tongzipo Road, Changsha 410013, China; zengqian02@csu.edu.cn (Q.Z.); liaominlin120@csu.edu.cn (M.-L.L.); 227811043@csu.edu.cn (Y.-Y.L.); 247811054@csu.edu.cn (S.L.); 257811045@csu.edu.cn (G.Y.); 2 School of Nursing, Ningxia Medical University, 1160 Shengli Street, Yinchuan 750003, China; chongmeihuang@nxmu.edu.cn (C.-M.H.); liuminhui@nxmu.edu.cn (M.-H.L.)

**Keywords:** pulmonary fibrosis, lathyrol, PPARγ, TGF-β/Smad signaling pathway

## Abstract

Idiopathic pulmonary fibrosis is a chronic, progressive, interstitial lung disease for which specific and effective drug therapies are still lacking. Lathyrol is a diterpene compound with broad pharmacological activities that can be extracted from the traditional Chinese medicine *Leptochloa chinensis* (L.) Nees. To investigate the anti-pulmonary fibrosis effect of lathyrol and its underlying mechanism. In vivo, a mouse model of pulmonary fibrosis was induced by bleomycin, treated with intraperitoneal injections of lathyrol. In vitro, myofibroblast conversion was induced in three fibroblast cell lines by stimulating them with TGF-β1, followed by treatment with lathyrol. Transcriptomic analysis was performed to assess the regulation of signaling pathways and gene expression patterns modulated by lathyrol. The effects of lathyrol on PPARγ activation, as well as on the nuclear translocation and ubiquitination of phosphorylated Smad3, were examined. The interaction among Nedd4, PPARγ, and phosphorylated Smad3 was detected. In vivo, lathyrol ameliorated pathological fibrosis in the lungs of mice with pulmonary fibrosis and this effect was blocked by a PPARγ inhibitor. In vitro, lathyrol inhibited the transdifferentiation of fibroblasts into myofibroblasts, and these effects were suppressed by either inhibiting PPARγ activation or specifically silencing the PPARγ gene. Lathyrol inhibited the nuclear translocation of phosphorylated Smad3 and promoted its ubiquitination, while also enhancing the interaction among Nedd4, PPARγ, and phosphorylated Smad3. These effects were abolished following the specific silencing of either PPARγ or Nedd4. In conclusion, Lathyrol inhibits myofibroblast transformation by suppressing TGF-β/Smad pathway activation through PPARγ activation, thereby exerting its anti-pulmonary fibrosis effects.

## 1. Introduction

Idiopathic pulmonary fibrosis (IPF) is a chronic, progressive, fibrotic lung disease of unknown etiology [1], characterized by remodeling of the pulmonary interstitium, distal airways, and alveolar spaces [2]. IPF is an age-related disease with sharply increasing incidence with age, reflected by a median survival of only 3–5 years [3]. With the progressive aging of the global population, the clinical and socioeconomic burden of the disease on both patients and healthcare systems is steadily increasing [4]. Currently, the drugs used clinically to treat the disease are pirfenidone and nintedanib that can only delay the disease progression and carry multiple side effects [5]. Therefore, exploring new drugs for the effective treatment of IPF is urgently needed.

Although the pathogenesis of IPF remains incompletely understood, it is well-established that multiple cell types and cytokines are involved, including myofibroblasts. Myofibroblasts are critically involved in the development and progression of IPF, where their aberrant activation and accumulation lead to excessive collagen deposition, destruction of alveolar structure, and ultimately irreversible loss of lung function [6]. The differentiation and proliferation of myofibroblasts are primarily regulated by transforming growth factor-β (TGF-β). This cytokine possesses multiple physiological functions, including immunoregulation, tissue repair, pro-fibrotic effect, and cell proliferation [7,8,9,10]. In the lung tissue of IPF patients, the expression of TGF-β is significantly upregulated, driving myofibroblast proliferation and differentiation [11]. The pro-fibrotic effect of TGF-β in IPF primarily depends on downstream Smad and non-Smad pathways, particularly the TGF-β/Smad canonical pathway. Upon binding to its receptors, TGF-β1 triggers the phosphorylation of Smad2/3, which subsequently associate with Smad4 to form a heteromeric signaling complex [12,13,14]. Following its translocation into the nucleus, this complex regulates the transcription of target genes associated with pulmonary fibrosis. In vitro, inhibition of the TGF-β/Smad pathway significantly suppresses the transformation of fibroblasts into myofibroblasts [15,16]. In vivo, inhibition of this pathway suppresses pulmonary fibrosis induced by multiple factors [17,18,19,20]. Therefore, TGF-β-induced fibroblast differentiation and the associated TGFβ/Smad pathway represent key therapeutic targets for IPF.

Peroxisome proliferator-activated receptor gamma (PPARγ) is a ligand-activated nuclear transcription factor that plays crucial roles in lipid metabolism, inflammation suppression, cell differentiation, proliferation inhibition, and apoptosis induction [21,22,23]. Owing to its broad spectrum of activity and capacity to bind both natural and synthetic ligands, PPARγ has emerged as a promising therapeutic target for various inflammatory and pulmonary fibrotic diseases. In vivo, PPARγ agonists such as lanifibranor and curcumin act by activating the PPARγ pathway to inhibit pulmonary fibrosis induced by bleomycin (BLM), SiO_2_, or paraquat [24,25,26,27,28]. In vitro, ligand binding with agents such as metformin activates PPARγ, which in turn exerts anti-fibrotic effect by promoting the transdifferentiation of myofibroblasts into adipocytes and inhibiting the proliferation and differentiation of fibroblasts [29,30]. Although PPARγ activation has been demonstrated to suppress pulmonary fibrosis in vivo and in vitro, its detailed molecular mechanism remains poorly elucidated. Activation of PPARγ has also been shown to inhibit the TGF-β/Smad pathway which is a key pathway in pulmonary fibrosis. However, the precise molecular mechanism underlying this inhibitory effect remains to be fully elucidated. Therefore, exploring the detailed mechanism by which PPARγ activation suppresses pulmonary fibrosis is equally significant.

Lathyrol is a diterpene that can be extracted from the plant named *Leptochloa chinensis* (L.) Nees, exhibits multiple pharmacological effects including anti-inflammatory and anti-tumor activities [31,32,33]. Although lathyrol has not yet been demonstrated to possess anti-pulmonary fibrosis effect, another diterpenoid compound from *Leptochloa chinensis* (L.) Nees, ingenol, has been demonstrated efficacy in ameliorating silica-induced pulmonary fibrosis [34]. Additionally, studies indicate that lathyrol can inhibit the TGF-β/Smad signaling pathway and suppress the expression of EMT-associated proteins, such as N-cadherin, β-catenin, and vimentin [32,35]. In summary, we hypothesize that lathyrol may also possess potent anti-pulmonary fibrotic effect. In this study, we investigated the therapeutic effect of lathyrol on bleomycin-induced pulmonary fibrosis in mice and elucidate its underlying mechanism.

## 2. Results

### 2.1. Lathyrol Reduced BLM-Induced Pulmonary Fibrosis in Mice

**Figure 1:**
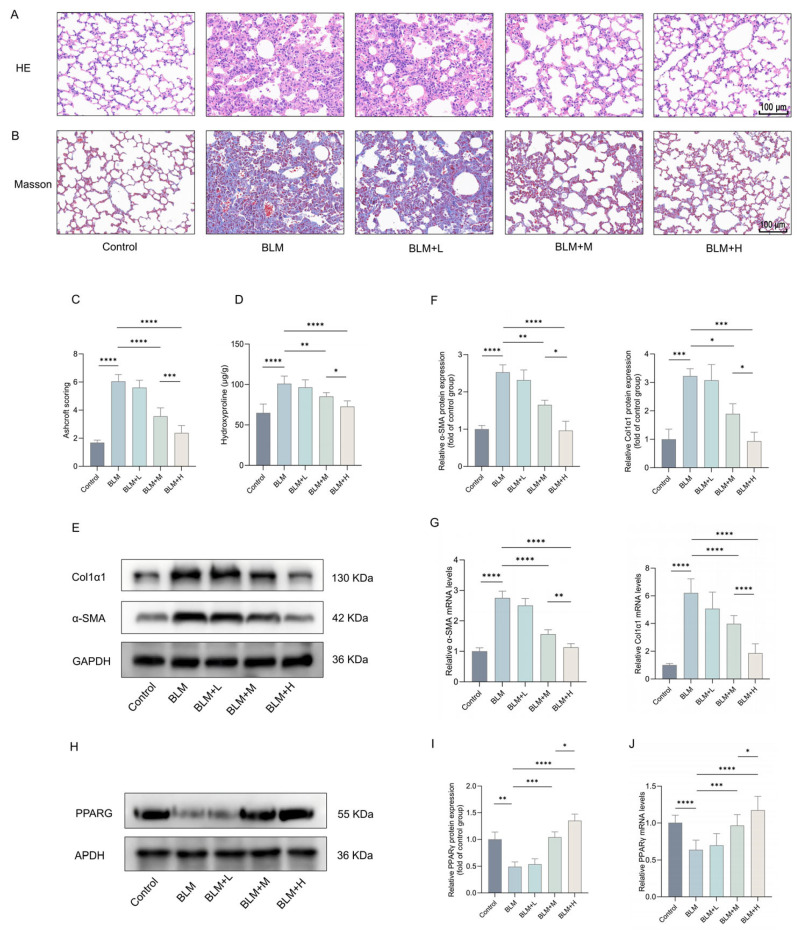
Anti-pulmonary fibrosis effect of lathyrol. (**A**,**B**) HE and Masson staining of mouse lung tissues (scale bar: 100 um). (**C**) Ashcroft scoring assessment of pulmonary fibrosis severity. (**D**) Biochemical assay of hydroxyproline content in mouse lung tissues. (**E**,**F**) WB detection of α-SMA and Col1α1 protein expression in mouse lung tissues. (**G**) RT-qPCR detection of *α-SMA* and *Col1α1* mRNA expression in mouse lung tissues. (**H**,**I**) WB detection of PPARγ protein expression levels in mouse lung tissues. (**J**) RT-qPCR detection of *PPARγ* mRNA expression levels in mouse lung tissues. Control represents the control group, BLM represents the pulmonary fibrosis group, BLM + L represents the low-dose lathyrol treatment group, BLM + M represents the medium-dose lathyrol treatment group, and BLM + H represents the high-dose lathyrol treatment group. Data are expressed as mean ± standard deviation. *n* = 8 per group. * *p* < 0.05, ** *p* < 0.01, *** *p* < 0.001, **** *p* < 0.0001.

Lathyrol were administered intraperitoneally at varying concentrations to observe its direct anti-fibrotic efficacy. HE and Masson’s staining showed that treatment with medium and high doses of lathyrol improved extracellular matrix (ECM) deposition, the destruction of alveolar structure, and the thickening of alveolar walls in the lungs of BLM-induced fibrotic mice (Figure 1A,B). Similarly, Ashcroft scoring demonstrated a significant amelioration of these pathological changes after lathyrol treatment (Figure 1C). Treatment with medium and high doses of lathyrol also significantly reduced the hydroxyproline content in the lung tissues of fibrotic mice (Figure 1D). Western blotting (WB) and real-time quantitative reverse transcription polymerase chain reaction (RT-qPCR) analyses revealed that treatment with medium and high doses of lathyrol significantly reduced the expression levels of the fibrosis markers α-SMA and Col1α1 in the lung tissues of fibrotic mice (Figure 1E–G). Notably, the high dose of lathyrol exhibited a more pronounced anti-fibrotic effect compared to the medium dose. In summary, lathyrol effectively alleviates BLM-induced pulmonary fibrosis in mice. Other compounds derived from *Leptochloa chinensis* (L.) Nees, such as kaempferol-3-glucuronide and quercetin-3-glucuronide, have been reported to exert effects on PPARγ [36]. Therefore, to investigate the detailed mechanism underlying lathyrol’s anti-pulmonary fibrosis effect, we also examined PPARγ expression in the lung tissues of mice with pulmonary fibrosis. The results showed that PPARγ expression was significantly downregulated in the lung tissues of mice with pulmonary fibrosis compared to the control group, while treatment of medium and high doses of lathyrol could inhibit this change (Figure 1H–J). This suggests that anti-pulmonary fibrosis effect of lathyrol may be related to its influence on the PPARγ pathway.

### 2.2. Lathyrol Inhibited TGF-β1-Induced Fibroblast-to-Myofibroblast Transdifferentiation

**Figure 2:**
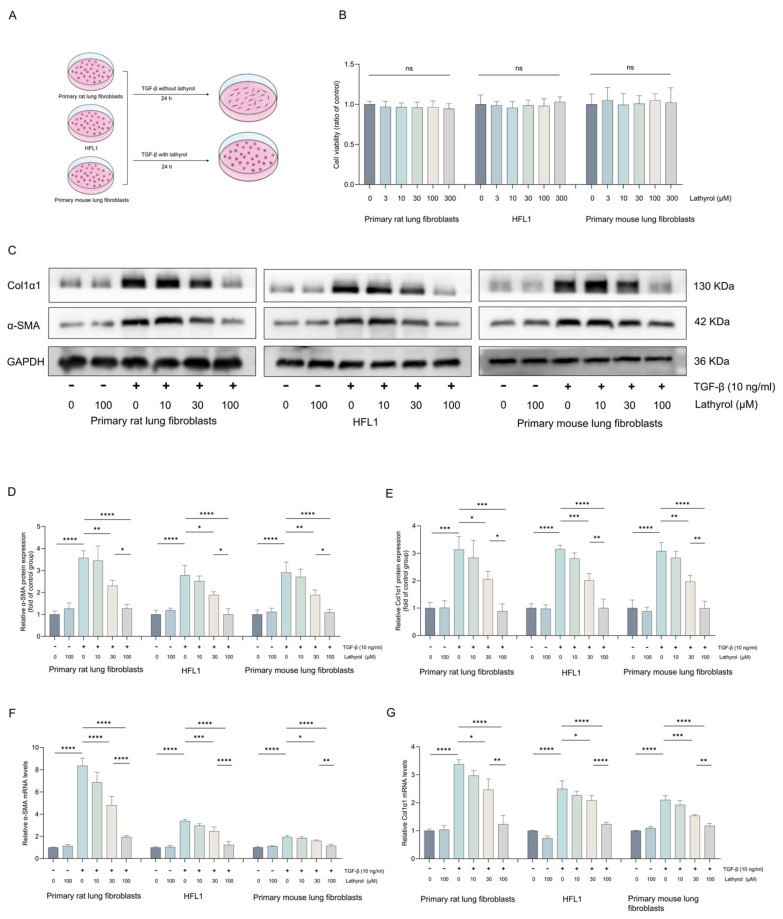
Anti-myofibroblast transforming effect of lathyrol. (**A**) Three types of fibroblasts were treated with lathyrol and/or TGF-β1. (**B**) Biochemical method detection of the effects of different concentrations of lathyrol in the three cell types. (**C**–**E**) WB detection of the protein expression levels of α-SMA and Col1α1 in the three cell types. (**F**,**G**) RT-qPCR detection of the mRNA expression levels of *α-SMA* and *Col1α1* in the three cell types. Data are presented as mean ± standard deviation. All experiments were independently repeated at least three times. ns represents no significance, * *p* < 0.05, ** *p* < 0.01, *** *p* < 0.001, **** *p* < 0.0001.

The differentiation of fibroblasts into myofibroblasts is a critical pathological change in the development of pulmonary fibrosis. To determine if anti-pulmonary fibrotic effect of lathyrol is mediated through the inhibition of fibroblast-to-myofibroblast differentiation, we used three distinct fibroblasts: primary mouse pulmonary fibroblasts, primary rat pulmonary fibroblasts, and the HFL1 (Figure 2A). Firstly, we assessed the cytotoxicity of lathyrol at a concentration range of 1–300 μM on all three cell types. The results showed that lathyrol did not significantly affect the viability of any of the cell types within this concentration range (Figure 2B). Cells then were treated with lathyrol for 24 h with or without TGF-β1. Both WB and RT-qPCR results confirmed that the treatment of lathyrol at 30 and 100 μM suppressed the TGF-β1-induced the expression of α-SMA and col1α1, with the 100 μM dose being more effective (Figure 2C–G). Collectively, these findings demonstrate that lathyrol effectively suppresses the differentiation of fibroblasts into myofibroblasts in vitro, suggesting that this may be a key mechanism contributing to its anti-pulmonary fibrosis effect.

### 2.3. Lathyrol Activated the PPARγ in Fibroblasts

**Figure 3:**
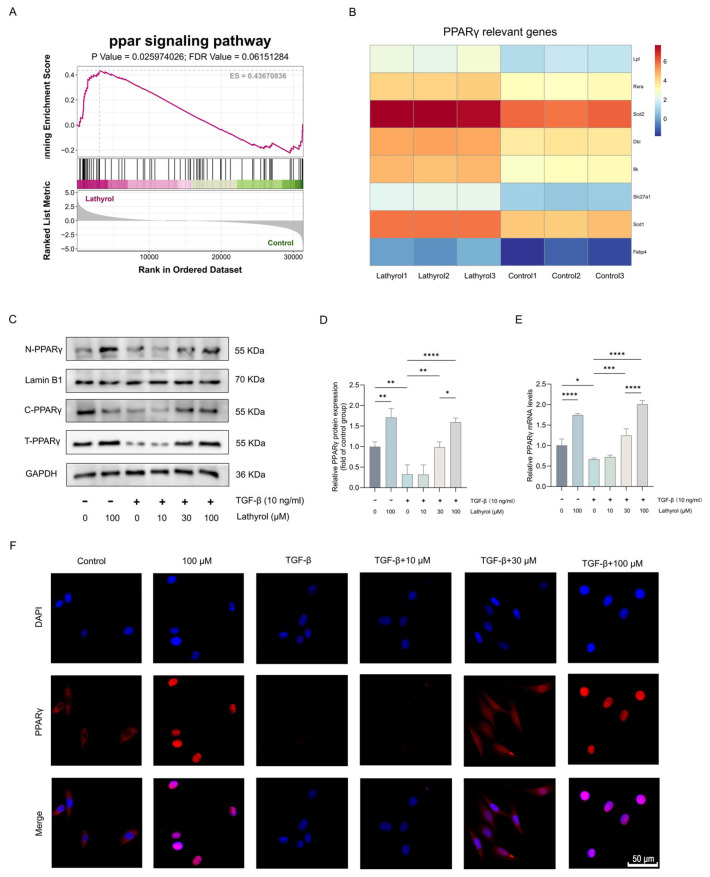
The activation effect of lathyrol on the PPARγ signaling pathway. (**A**) GSEA enrichment analysis after lathyrol treatment. (**B**) Differentially expressed PPARγ-related genes after lathyrol treatment. (**C**,**D**) WB detection of nuclear translocation and expression of PPARγ. (**E**) RT-qPCR detection of mRNA expression levels of *PPARγ*. (**F**) Immunofluorescence detection of nuclear translocation and expression levels of PPARγ (scale bar: 50 um). Data are presented as mean ± standard deviation. All experiments were independently repeated at least three times. * *p* < 0.05, ** *p* < 0.01, *** *p* < 0.001, **** *p* < 0.0001.

In vivo experiments demonstrated that lathyrol not only ameliorated BLM-induced pulmonary fibrosis in mice but also suppressed the reduction in PPARγ expression caused by pulmonary fibrosis. Furthermore, several other compounds derived from *Leptochloa chinensis* (L.) Nees have been shown to activate the PPARγ pathway [37,38,39]. To determine whether lathyrol activates the PPARγ pathway in pulmonary fibroblasts, lathyrol-treated primary mouse pulmonary fibroblasts were subjected to transcriptomic analysis. The results revealed that lathyrol significantly activated the PPAR signaling pathway and markedly increased the expression of multiple PPARγ-related genes. (Figure 3A,B). Lathyrol at 30 and 100 μM both significantly suppressed the TGF-β1-induced downregulation of PPARγ expression and markedly promoted its nuclear translocation (Figure 3C–F). In summary, these findings indicate that lathyrol activates the PPARγ pathway, suggesting a potential mechanism for its anti-myofibroblast transforming and anti-pulmonary fibrosis effects.

### 2.4. PPARγ Mediated the Inhibition of Myofibroblast Transformation by Lathyrol

**Figure 4:**
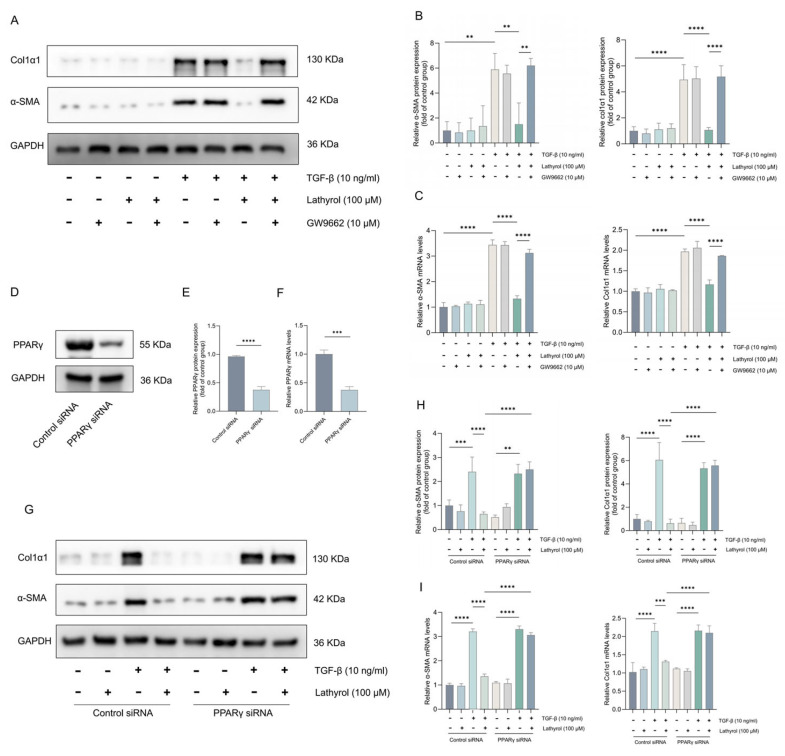
Lathyrol activated PPARγ to inhibit myofibroblast transformation. (**A**,**B**) WB detection of the protein expression levels of α-SMA and Col1α1 in cells. (**C**) RT-qPCR detection of the mRNA expression levels of *α-SMA* and *Col1α1*. (**D**–**F**) WB and RT-qPCR validation of PPARγ knockdown efficiency. (**G**,**H**) WB detection of the protein expression levels of α-SMA and Col1α1. (**I**) RT-qPCR detection of the mRNA expression levels of *α-SMA* and *Col1α1*. Data are expressed as mean ± standard deviation. All experiments were independently repeated at least three times. ** *p* < 0.01, *** *p* < 0.001, **** *p* < 0.0001.

To investigate whether lathyrol’s inhibition of myofibroblast differentiation is associated with its activation of the PPARγ pathway, we conducted further experiments using primary mouse fibroblasts. Cells were treated with the PPARγ antagonist GW9662 or transfected with PPARγ siRNA, and the subsequent role on lathyrol’s inhibition of myofibroblast differentiation was observed. WB and RT-qPCR revealed that the reduction in TGF-β1-induced α-SMA and Col1α1 expression by lathyrol was counteracted upon treatment with GW9662 (Figure 4A–C). Likewise, in cells transfected with PPARγ siRNA, the inhibitory effect of lathyrol on TGF-β1-induced α-SMA and Col1α1 expression was also abrogated (Figure 4D–I). Collectively, these findings demonstrate that lathyrol inhibits the TGF-β1-induced fibroblast-to-myofibroblast transition through the activation of PPARγ.

### 2.5. Lathyrol Inhibits the TGF-β/Smad Pathway by Activating PPARγ

**Figure 5:**
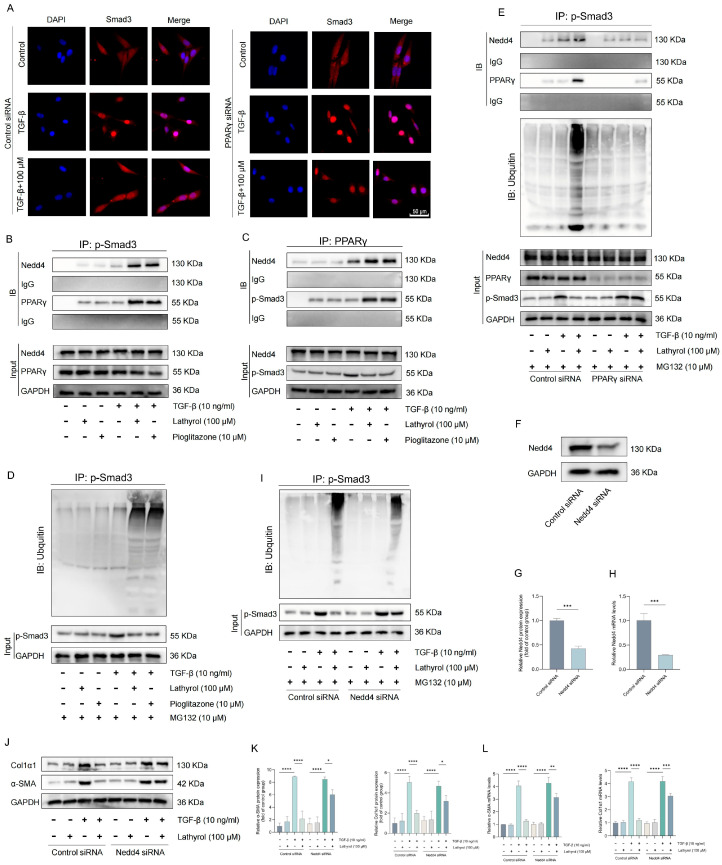
Lathyrol inhibited p-Smad3 nuclear translocation and promoted its ubiquitination. (**A**) Immunofluorescence detection of Smad3 nuclear translocation (scale bar: 50 um). (**B**,**C**) Co-IP detection of PPARγ, p-Smad3 and Nedd4 interactions. (**D**) Co-IP detection of p-Smad3 ubiquitination level. (**E**) Co-IP detection of p-Smad3 interactions with PPARγ and Nedd4 after PPARγ silencing and the ubiquitination level of p-Smad3. (**F**–**H**) WB and RT-qPCR validation of Nedd4 silencing efficacy. (**I**) Co-IP detection of p-Smad3 ubiquitination level after Nedd4 silencing. (**J**,**K**) WB detection of α-SMA and Col1α1 protein expression levels after Nedd4 silencing. (**L**) RT-qPCR detection of mRNA expression levels of *α-SMA* and *Col1α1* after Nedd4 silencing. Data are presented as mean ± standard deviation. All experiments were independently repeated at least three times. * *p* < 0.05, ** *p* < 0.01, *** *p* < 0.001, **** *p* < 0.0001.

We have demonstrated that lathyrol inhibits myofibroblast transformation by activating PPARγ. However, the mechanism by which PPARγ activation suppresses myofibroblast transformation and inhibits pulmonary fibrosis remains unclear. Previous studies have shown that activated PPARγ can directly bind to phosphorylated Smad3 (p-Smad3), thereby inhibiting its nuclear translocation and suppressing the TGF-β/Smad signaling pathway [40]. This mechanism may, therefore, underlie the inhibitory effect of lathyrol on myofibroblast transformation and pulmonary fibrosis. Immunofluorescence analysis revealed that lathyrol also inhibited the TGF-β-induced nuclear translocation of Smad3 (Figure 5A). Furthermore, lathyrol also promote the direct binding of PPARγ to p-Smad3, an effect similar to that of pioglitazone (Figure 5B,C). Thus, lathyrol likely exerts its effect on the TGF-β/Smad pathway by inhibiting p-Smad3 nuclear translocation through PPARγ activation. Although lathyrol promotes the binding of PPARγ to p-Smad3 in the cytoplasm, it remains unclear what biological processes occur after this interaction. It is established that activated PPARγ can combine with multiple proteins; some function as coactivators to enhance the activation, whereas others mediate distinct biological functions. For instance, Nedd4, an E3 ubiquitin ligase predominantly localized in the cytoplasm, is able to bind PPARγ and modulate its expression and function [41]. Therefore, we hypothesize that upon activation, PPARγ may interact with other proteins while bound to p-Smad3 in the cytoplasm to mediate relevant effects. According to the Co-IP experiments, we found that both lathyrol and pioglitazone promote the ubiquitination of p-Smad3 (Figure 5D). Furthermore, both compounds enhanced the co-binding of Nedd4 to PPARγ and p-Smad3 (Figure 5B,C). Notably, these effects were abolished upon PPARγ silencing (Figure 5E). Therefore, lathyrol may also inhibit the TGF-β/Smad pathway by promoting the ubiquitin-mediated degradation of p-Smad3, a mechanism likely closely associated with Nedd4. Indeed, we found that specific silencing of Nedd4 suppressed both lathyrol-induced p-Smad3 ubiquitination caused by lathyrol and its inhibition of myofibroblast transformation (Figure 5F–L), confirming that Nedd4 mediates the suppression of the TGF-β/Smad pathway via ubiquitination of p-Smad3. In summary, following PPARγ activation, lathyrol not only directly inhibits the nuclear translocation of p-Smad3 but also suppresses the TGF-β/Smad signaling pathway by promoting the ubiquitination of p-Smad3. This may represent a key mechanism by which lathyrol inhibits myofibroblast transformation and pulmonary fibrosis.

### 2.6. PPARγ Mediated the Anti-Pulmonary Fibrosis Effects of Lathyrol In Vivo

**Figure 6:**
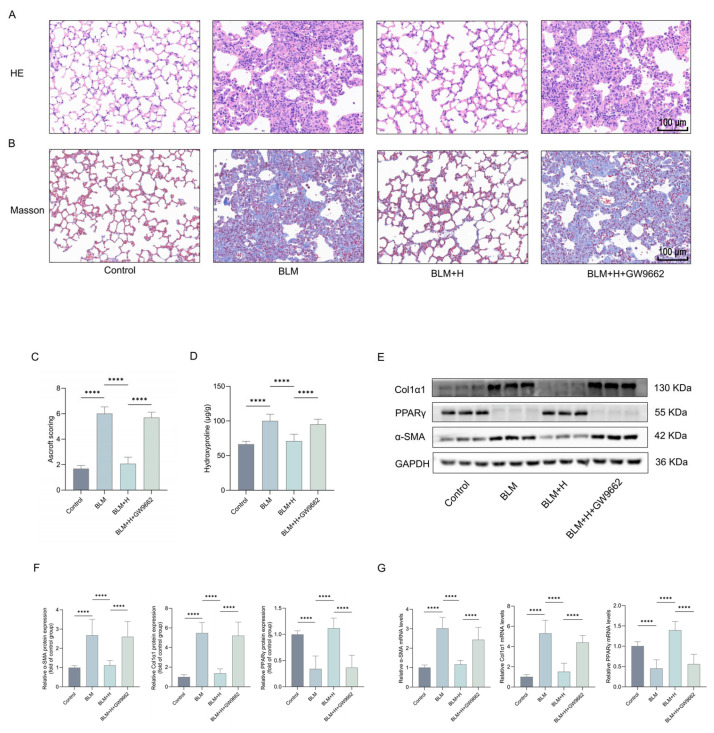
GW9662 inhibited the anti-pulmonary fibrosis effect of lathyrol in vivo. (**A**,**B**) HE and Masson staining of mouse lung tissues (scale bar: 100 um). (**C**) Ashcroft scoring assessment of pulmonary fibrosis severity. (**D**) Biochemical assay of hydroxyproline content in mouse lung tissues. (**E**,**F**) WB detection of α-SMA, Col1α1 and PPARγ protein expression in mouse lung tissues. (**G**) RT-qPCR detection of *α-SMA*, *Col1α1* and *PPARγ* mRNA expression levels in mouse lung tissues. Control represents the control group, BLM represents the pulmonary fibrosis group, BLM + H represents the high-dose lathyrol treatment group, and BLM + H + GW9662 represents the GW9662 and lathyrol co-treatment group. Data are expressed as mean ± standard deviation. *n* = 8 per group. **** *p* < 0.0001.

Our in vitro results indicate that PPARγ mediated lathyrol’s anti-myofibroblast transformation effects. To further investigate whether lathyrol’s inhibition of pulmonary fibrosis correlates with its activation of PPARγ, mice were pretreated with the PPARγ antagonist GW9662 before the intraperitoneal administration of lathyrol. HE and Masson’s staining, coupled with ashcroft scoring, demonstrated that GW9662 inhibited the ameliorative effects of lathyrol on ECM deposition, alveolar structural disruption, and alveolar wall thickening in the lungs of pulmonary fibrosis mice (Figure 6A–C). Furthermore, GW9662 treatment also reversed lathyrol’s reduction in hydroxyproline content in lung tissues (Figure 6D). Consistent with this, GW9662 also counteracted the suppressive effect of lathyrol on the protein and mRNA expression levels of α-SMA, Col1α1, and PPARγ in mice with pulmonary fibrosis (Figure 6E–G). Taken together, these findings strongly suggest that PPARγ mediates the anti-pulmonary fibrosis effect of lathyrol in vivo.

## 3. Discussion

In this study, we first identified that lathyrol suppresses the fibroblast-to-myofibroblast differentiation by activating PPARγ to inhibit the TGF-β/Smad pathway, thereby alleviating BLM-induced pulmonary fibrosis in mice. Moreover, we have demonstrated for the first time that PPARγ activation suppresses the TGF-β/Smad pathway not only by directly inhibiting the nuclear translocation of p-Smad3, but also by promoting the ubiquitination of p-Smad3. This mechanism underlies the anti-myofibroblast transformation and anti-pulmonary fibrosis effects of lathyrol.

Following tracheal injection of BLM in mice, fibrosis began to develop in mouse lung tissues on day 15, with extensive fibroblast proliferation and differentiation into myofibroblasts. This was accompanied by a progressive increase in ECM deposition, which peaked at day 28. We administered lathyrol via intraperitoneal injection in mice from days 15 to 28, the results indicated that lathyrol treatment significantly attenuated the expression of key fibrotic markers and ameliorated the pathological changes in lung tissue architecture, demonstrating that lathyrol directly ameliorates BLM-induced pulmonary fibrosis in mice. The intraperitoneal dosage of lathyrol was determined based on a review of prior studies [32,33]. While a separate toxicological study for high-dose lathyrol was not conducted, no significant adverse effects were observed in mice in other studies or in our present study. Furthermore, we did not compare the therapeutic effect of lathyrol and that of current clinical anti-pulmonary fibrosis drugs pirfenidone and nintedanib in treatment of mice with pulmonary fibrosis. These limitations of the present study should be addressed in future research. In addition, we ultimately selected α-SMA and Col1α1 to reflect lathyrol’s anti-fibrotic activity. Consequently, the failure to incorporate MMPs/TIMPs, pro-inflammatory cytokines, and EMT markers is a recognized limitation of this study. Therefore, our future research will focus on in-depth mechanistic investigations of these pathways, specifically targeting IL-6, TNF-α, the MMP/TIMP balance, and EMT markers such as E-cadherin and N-cadherin, to construct a more comprehensive signaling profile.

IPF is pathologically characterized by the excessive accumulation of fibrous tissue within the lung parenchyma, replacement of healthy tissue by altered ECM, and destruction of alveolar architecture. Myofibroblast transdifferentiation represents one of the primary pathogenic mechanisms in IPF. This process is initiated by repeated micro-injuries to alveolar type II epithelial cells, which results in increased secretion of TGF-β, triggering the extensive proliferation and activation of fibroblasts [42]. As key effector cells in IPF, persistently activated myofibroblasts produce excessive amounts of collagen. This leads to the disruption of normal lung architecture and aberrant tissue repair, ultimately driving the progression of pulmonary fibrosis. Studies indicate that inhibiting the activation of myofibroblasts can effectively reduce pulmonary fibrosis [43]. The TGF-β family is one of the key factors in suppressing myofibroblast activation. As a pro-fibrotic cytokine, excessive activation of TGF-β promotes the differentiation of fibroblasts into myofibroblasts. To determine whether the anti-pulmonary fibrosis effect of lathyrol is associated with its inhibition of myofibroblast transformation, three different types of fibroblasts undergoing TGF-β1-induced myofibroblast transformation were treated with lathyrol. The results showed that lathyrol significantly inhibited the TGF-β1-induced myofibroblast transformation of the three fibroblast types. This inhibitory effect on myofibroblast transformation may also be key to the anti-pulmonary fibrosis effect of lathyrol.

Peroxisome proliferator-activated receptors (PPARs) are nuclear receptors with ligand-activated transcription factor functions, and PPARγ is one of its isoforms. As a heterodimer, PPARγ can bind to the peroxisome proliferator response element within the promoter region with the retinoic acid X receptor, thereby regulating gene expression [44]. Flavonoids from *Leptochloa chinensis* (L.) Nees, such as kaempferol-3-glucuronide and quercetin-3-glucuronide, can activate the PPARγ [36]. Based on this, we also investigated whether lathyrol could activate PPARγ and whether its anti-pulmonary fibrosis and anti-myofibroblast transformation effects were also mediated by its interaction with PPARγ. Transcriptomic analysis revealed that lathyrol significantly activated the PPAR signaling pathway and markedly promoted the expression of PPARγ relevant genes. And our results directly confirmed the activation of the PPARγ pathway by lathyrol. In vitro, inhibiting PPARγ activation or specifically silencing PPARγ attenuated the anti-myofibroblast transformation effect of lathyrol. In vivo, PPARγ inhibitor similarly attenuated the anti-pulmonary fibrosis effect of lathyrol. These findings indicate that lathyrol also functions via the activation of PPARγ, and this action is crucial for its anti-pulmonary fibrosis and anti-myofibroblast transformation effects. Although our study demonstrates that lathyrol exerts anti-pulmonary fibrosis and anti-myofibroblast transformation effects by activating PPARγ, it was not directly determined whether it functions as a direct ligand for PPARγ. This warrants further investigation.

PPARγ plays a crucial role in IPF progression, and its anti-fibrotic effects are mediated through multiple mechanisms, including modulation of the Smad pathway to inhibit the pro-fibrotic signal of TGF-β [45]. The role of TGF-β in IPF primarily depends on downstream TGF-β/Smad classical and non-classical pathways. The canonical pathway is the primary mechanism driving the onset and progression of IPF. TGF-β1 phosphorylates Smad2/3, leading to the formation of multiple Smad proteins complexes and their subsequent nuclear translocation, which results in aberrant ECM deposition and tissue remodeling. This study reveals that, like various other PPARγ agonists, lathyrol also inhibits the TGF-β/Smad pathway. Calvier et al. indicated that the inhibition of the TGF-β/Smad pathway by PPARγ activation may be mediated by the direct binding of PPARγ to p-Smad3, thereby preventing its nuclear translocation [40]. Therefore, the effect of lathyrol on TGF-β1-induced nuclear translocation of Smad3 protein was also examined. The results indicated that lathyrol directly promotes the binding of PPARγ to p-Smad3, thereby inhibiting Smad3 nuclear translocation. This mechanism likely represents a pathway by which lathyrol suppresses the TGF-β/Smad pathway and myofibroblast transformation via PPARγ activation. PPARγ is a receptor capable of binding multiple ligands, and different ligands may exert distinct biological effects upon binding. For example, PPARγ interacts with Nedd4 through its hinge and ligand-binding domains, and with Nedd4l via a conserved PPXY-WW binding motif [46,47]. Nedd4 regulates stability of PPARγ and PPARγ activation promotes the mRNA and protein expression of Nedd4 [48,49]. Therefore, upon activating PPARγ, lathyrol may not only facilitate the binding of PPARγ to p-Smad3 but also interact with other molecules, thereby modulating the state or function of p-Smad3. In our results, we found that lathyrol promotes the ubiquitination of p-Smad3. We hypothesized that this process may involve ubiquitin ligases that bind to activated PPARγ. Accordingly, we investigated the effect of lathyrol on Nedd4, a ubiquitin ligase that directly binds to PPARγ. The results showed that both lathyrol and pioglitazone promoted the binding of the PPARγ and p-Smad3 complex to Nedd4, and this effect was suppressed by the specific silencing of PPARγ. To further validate the relevance of this effect to the anti-myofibroblastic activity of lathyrol, the nedd4 gene was specifically silenced. The results showed that the anti-myofibroblastic activity of lathyrol and its promotion of p-Smad3 ubiquitination were significantly suppressed, suggesting that lathyrol inhibits the TGF-β/Smad pathway by promoting the ubiquitination of p-Smad3 through PPARγ activation. Nedd4 and Nedd4l are both HECT-domain E3 ubiquitin ligases that regulate cellular functions by mediating the fate of substrate proteins [50]. Nedd4l has been demonstrated to specifically recognize the Thr-Pro-Tyr motif within the linker region, promoting the polyubiquitination and subsequent degradation of Smad3 [51]. And knockout of nedd4l accelerates the progression of pulmonary fibrosis [52,53]. Nedd4 has been shown to participate in TGF-β signaling in hepatocellular carcinoma by regulating Smad3, though the detailed mechanism remains unclear [54]. Our study further demonstrates that nedd4 inhibits the TGF-β/Smad pathway by promoting the ubiquitination of p-Smad3 in a model of TGF-β1-induced myofibroblast transformation, suggesting that Nedd4 could also serve as a potential therapeutic target for pulmonary fibrosis. Furthermore, PPARγ could competitively binding to p300, thereby suppressing Smad-mediated transcriptional responses by preventing p300 recruitment, which results in the inhibition of TGF-β-induced gene expression [55,56]. However, our study did not specifically examine whether lathyrol exerts this effect, which represents a key limitation. We plan to address this aspect in our future research.

Although our study demonstrated the anti-fibrotic effects of lathyrol in a bleomycin-induced mouse model of pulmonary fibrosis, its therapeutic efficacy in other fibrotic models, such as those induced by SiO_2_ and paraquat, requires further investigation. Furthermore, given that IPF is a chronic and progressive disease, the absence of long-term follow-up in our study precludes a direct assessment of lathyrol’s sustained therapeutic effects. Additionally, our in vitro experiments also failed to demonstrate lathyrol’s inhibitory effect on TGF-β1-induced myofibroblastic transformation in fibroblasts derived from IPF patients. These points constitute the primary limitations of our current work, which we intend to address in our future research.

Lathyrol is an active diterpenoid compound, could be extracted from Chinese medicine *Leptochloa chinensis* (L.) Nees. *Leptochloa chinensis* (L.) Nees exhibits a wide range of pharmacological effects, primarily including diuretic, anti-inflammatory, antibacterial, antitumor, and antioxidant activities [57]. However, no previous studies have documented a correlation between the pharmacological effects of *Leptochloa chinensis* (L.) Nees and the activation of PPARγ. Nevertheless, active components from *Leptochloa chinensis* (L.) Nees, such as the aesculin, can activate PPARγ [58]. In our study, we discovered that lathyrol exerts anti-pulmonary fibrosis effect by activating PPARγ. Therefore, whether the other pharmacological effects of lathyrol and the pharmacological effects other compounds extracted from *Leptochloa chinensis* (L.) Nees are also associated with PPARγ activation warrants further investigation.

## 4. Materials and Methods

### 4.1. Experimental Animals and Pulmonary Fibrosis Models

Eight-week-old male C57BL/6 mice and SD rats were purchased from the Animal Department of Central South University. After a one-week acclimatization period in a specific pathogen-free barrier facility with appropriate temperature and humidity, the mice were randomly allocated into control and bleomycin-induced model group. Following anesthesia with sodium pentobarbital, mice in the bleomycin-induced model group received an intratracheal instillation of 50 μL of bleomycin solution (Nippon Kayaku, Tokyo, Japan) at a dose of 3 mg/kg. Similarly, the control group received an equivalent volume of sterile saline via the same intratracheal instillation procedure. Lathyrol (Solarbio, Beijing, China) was dissolved in a vehicle composed of 5% dimethyl sulfoxide (Macklin, Shanghai, China), 40% polyethylene glycol 300 (Macklin), 5% Tween 80 (Macklin), and 50% sterile saline.

Plan one: To investigate the in vivo anti-pulmonary fibrosis effects of lathyrol. Of 90 mice, 10 were randomly allocated to the control group, while the remaining 80 were used to establish the pulmonary fibrosis model. On day 14, the BLM-treated mice were divided into four groups of equal size. From days 15 to 28, the control and BLM model groups were administered the vehicle via intraperitoneal injection, while the low-dose group received 1 mg/kg lathyrol, the medium-dose group received 5 mg/kg lathyrol, and the high-dose group received 20 mg/kg lathyrol. On day 29, all mice were euthanized under sodium pentobarbital anesthesia, and lung tissues were collected for subsequent studies.

Plan Two: To validate that PPARγ mediates the in vivo anti-pulmonary fibrosis effects of lathyrol. Of 70 mice, 10 were randomly allocated to the control group, while the remaining 60 were used to establish the pulmonary fibrosis model. On day 14, the BLM-treated mice were divided into three groups of equal size. From days 15 to 28, the control and bleomycin-induced model groups were administered the vehicle via intraperitoneal injection. The high-dose group received intraperitoneal injections of lathyrol at 20 mg/kg. The GW9662 group was pretreated with an intraperitoneal injection of GW9662 (Sigma, St. Louis, MO, USA) at 1 mg/kg. 30 min later, these mice received an intraperitoneal injection of lathyrol at 20 mg/kg. On day 29, all mice were euthanized under sodium pentobarbital anesthesia, and lung tissues were collected for subsequent studies.

### 4.2. Hematoxylin and Eosin (HE) Staining, Masson’s Trichrome Staining, and Ashcroft Scoring

Mouse lung tissues were fixed in 4% paraformaldehyde, embedded in paraffin, and subsequently sectioned. Following deparaffinization, the sections were stained with dye solution (Servicebio, Wuhan, China) and then mounted. The Ashcroft scoring system was used to assess the severity of pulmonary fibrosis. In brief, the extent of pulmonary fibrosis in the HE-stained sections was independently assessed by two researchers under the microscope. The degree of fibrosis was scored according to established criteria, and the mean score was calculated.

### 4.3. Hydroxyproline Assay

Approximately 40 mg of mouse lung tissue was weighed. Tissue was mixed with saline at a weight-to-volume ratio of 1:9 (g/mL) and subsequently homogenized twice at 60 Hz for 60 s each using a high-speed tissue grinder (KZ-II, Servicebio). Then, 1 mL of alkaline hydrolysis solution was added, the mixture was thoroughly mixed, and it was hydrolyzed in a 95 °C water bath for 20 min. Each tube was cooled under running water and set aside for subsequent analysis. Following the instruction of the hydroxyproline assay kit (Nanjing Jiancheng Biotechnology Institute, Nanjing, China), reagents were sequentially added and thoroughly mixed. The homogenate was then incubated at 60 °C for 15 min. After cooling, the sample was centrifuged at 3500 rpm for 10 min. The resulting supernatant was transferred to a 96-well plate, and its absorbance was measured at 550 nm using an enzyme-linked immunosorbent assay reader (Varioskan LUX, Thermo Fisher Scientific, Waltham, MA, USA). Then, the hydroxyproline content was calculated based on the absorbance values.

### 4.4. RNA Extraction and RT-qPCR

After lysing mouse lung tissue or cells with Trizol reagent (Accurate Biology, Changsha, China), total RNA was extracted and purified using an RNA extraction kit (Accurate Biology). Then the purified RNA was eluted with enzyme-free water, and its concentration was quantified using a microplate reader. Reverse transcription was performed to cDNA from the RNA, according to the instruction of the reverse transcription kit (Accurate Biology). The reaction program was as follows: 37 °C for 15 min, followed by 85 °C for 5 s. Subsequently, real-time quantitative PCR was conducted using the cDNA as a template, according to the RT-qPCR kit (novoprotein, Nanjing, China) protocol. The RT-qPCR cycling conditions were: 95 °C for 2 min, followed by 40 cycles of 95 °C for 3 s and 60 °C for 30 s. A melting curve analysis was then performed from 60 °C to 95 °C. The primer sequences (Sangon Biotech, Shanghai, China) used in this study are listed in Table 1.

### 4.5. WB

Total proteins were extracted from mouse lung tissues or cells using RIPA lysis buffer (Solarbio) containing protease and phosphatase inhibitors (Solarbio). For adherent cells, 100 μL of RIPA lysis buffer was added to each well of a six-well plate. For mouse lung tissue, RIPA lysis buffer was added at a weight-to-volume ratio of 1:9 (g/mL). The samples were incubated on ice for 30 min and then centrifuged at 12,000 rpm for 15 min at 4 °C. The resulting supernatant was transferred to a new centrifuge tube, and the protein concentration was subsequently measured using a BCA assay kit (Cwbio, Taizhou, China). Proteins were denatured by heating the mixture in a 100 °C water bath for 15 min after an equal volume of loading buffer was added. The loading volume was adjusted based on the protein concentration, and the samples were subjected to 10% SDS-polyacrylamide gel electrophoresis (BIO-RAD, Hercules, CA, USA). Following electrophoresis, the proteins were transferred to PVDF membranes using ice-water bath transfer (constant current, 250 mA for 150 min; BIO-RAD). After transfer, the PVDF membranes were incubated in 5% non-fat milk at room temperature for 2 h to block non-specific binding. The membranes were incubated overnight at 4 °C with the antibody solution selected for the target proteins. The next day, the PVDF membranes were incubated at room temperature for 2 h with solution of HRP-conjugated goat anti-rabbit or anti-mouse IgG (Elabscience, Wuhan, China). Proteins expression were visualized using an imaging system (GeneGnome XRQ, Syngene, Cambridge, UK), and the images were captured. The antibodies used in this study are shown in Table 2.

### 4.6. Cell Extraction and Culture

Following euthanasia, the thoracic cavity of mice/rats was exposed to retrieve the lung tissues. The harvested lung tissues were washed with PBS containing 1% penicillin-streptomycin, minced, and subsequently placed in a 1 mg/mL solution of type I collagenase. The solution was incubated in a 37 °C water bath for 1 h and then centrifuged at 1500 rpm for 10 min, and then was resuspended and subsequently filtered through a 70 μm cell strainer. The tissues were then lysed on ice for 5 min using red blood cell lysis buffer, filtered through a 40 μm cell strainer, and resuspended in DMEM/F12 medium supplemented with 1% penicillin-streptomycin and 15% Fetal Bovine Serum (Procell, Wuhan, China). The isolated cells were cultured in a humidified incubator at 37 °C with 5% CO_2_. For subsequent experiments, primary mouse/rat lung fibroblasts between passages 3 and 7 were utilized. Human fetal lung fibroblasts (HFL1; Procell) were cultured in Ham’s F-12K medium (Procell) containing 1% penicillin-streptomycin and 10% fetal bovine serum, and maintained at 37 °C in a humidified incubator with 5% CO_2_.

### 4.7. Cell Viability Assay

The effect of various concentrations of lathyrol on cell viability were evaluated using a Cell Counting Kit-8 (CCK-8) assay (Elabscience). After trypsinization, cells were centrifuged, resuspended, and counted. The cells suspension was then adjusted to a density of 5 × 10^3^–5 × 10^4^ cells/mL and seeded into 96-well plates. After cells adhered, they were incubated with different concentrations of lathyrol for 24 h. Following PBS washing, CCK-8 reagent was added and the plate was incubated for 30 min at 37 °C, after which the absorbance at 450 nm was measured using a microplate reader.

### 4.8. RNA-Seq and Data Analysis

The RNA libraries were sequenced using the Illumina Novaseq™ 6000 platform (LC-Bio-Technology CO., Ltd., Hangzhou, China). Transcriptome assembly and annotation protocols were provided by LC Bio-Technology Co., Ltd.. Briefly, total RNA was extracted using TRIzol reagent (Invitrogen, Waltham, MA, USA) following the manufacturer’s instructions. And mRNA was purified from total RNA using Dynabeads Oligo (dT) (Thermo Fisher Scientific). Then the cleaved RNA fragments were reverse-transcribed to create the cDNA libraries with average insert sizes of 300 ± 50 bp. The 2 × 150 bp paired-end sequencing (PE150) was performed on an Illumina Novaseq 6000. And bioinformatic analysis was performed using the OmicStudio tools (https://www.omicstudio.cn/tool, accessed on 18 August 2025).

### 4.9. Immunofluorescence

Cells were cultured on coverslips and subjected to the specified treatments. According to the manufacturer’s instructions of tyramide signal amplification immunofluorescence kit (Aifang Biological, Changsha, China). The cells were then fixed with 5% paraformaldehyde for 15 min, permeabilized with 0.1% Triton X-100 for 15 min, and blocked with 5% goat serum for 30 min. Subsequently, the coverslips were incubated with PPARγ or Smad3 antibody solutions overnight at 4 °C. The next day, the cells were incubated with a polyclonal HRP-conjugated secondary antibody for 40 min at room temperature in the dark, followed by sequential incubation with a fluorescent dye for 10 min and DAPI for 15 min. After mounting, images were captured using a fluorescence microscope.

### 4.10. siRNA Transfection

According to the siRNA transfection protocol (Rebobio, Guangzhou, China), a 2 mL transfection mixture was consisted of 10 uL PPARγ or Nedd4 siRNA (Sangon Biotech) at 20 μM, 12 uL reagent and 120 uL buffer. The final concentration of siRNA in the mixture was 100 nM. Prior to transfection, cells at approximately 60% confluence were washed twice with PBS. The transfection mixture was then added to the cells and incubated for 24 h, after which the medium was replaced with normal culture medium.

### 4.11. Co-Immunoprecipitation (Co-IP)

Cells were lysed for 5 min on ice using RIPA lysis buffer containing protease inhibitors, and then centrifuged at 12,000 rpm for 10 min at 4 °C. Appropriate magnetic beads (MedChemExpress, Shanghai, China) were taken and washed four times with buffer. The beads were then incubated with either a p-Smad3 antibody, a PPARγ antibody, or an IgG antibody, and were incubated for 1 h at room temperature with rotation. The beads were washed four times with buffer, then incubated with the sample solution for 1 h at room temperature with rotation. Following a final wash, the beads were resuspended in 50 μL of loading buffer. After eluting the protein complex from the beads at 100 °C for 10 min, the samples were subjected to WB analysis.

### 4.12. Ubiquitination Assay

MG132 (Selleck Chemicals, Houston, TX, USA) were added 8 h prior to cell collection. Following the Co-IP protocol, whole-cell lysates were subjected to coupling using magnetic beads conjugated with p-Smad3 antibody. The eluate was collected as the final protein sample. The level of ubiquitinated p-Smad3 are then detected by WB using a polyclonal anti-ubiquitin antibody.

### 4.13. Statistical Analysis

Statistical analysis was performed using GraphPad Prism software (version 10.1). All data are expressed as the mean ± standard deviation. For comparisons among multiple groups, one-way ANOVA was used, followed by Tukey’s test for pairwise comparisons. For comparisons between two groups, an unpaired *t*-test was used. A *p*-value < 0.05 was considered statistically significant.

## 5. Conclusions

This study confirms the anti-pulmonary fibrosis effects of lathyrol and elucidates the mechanism by which PPARγ activation mediates these effects. Through the activation of PPARγ, lathyrol concurrently inhibits the nuclear translocation of p-Smad3 and promotes the formation of a complex among NEDD4, PPARγ, and p-Smad3 which results in the ubiquitination of p-Smad3, consequently suppressing the TGF-β/Smad signaling pathway. The inhibition of the TGF-β/Smad pathway via PPARγ activation mediates the suppression of fibroblast-to-myofibroblast transdifferentiation, which underlies the anti-pulmonary fibrosis effects of lathyrol.

## Figures and Tables

**Table 1:** The primer sequences.

Gene.	Forward [5′-3′]	Reverse [5′-3′]
Mouse GAPDH	GGTTGTCTCCTGCGACTTCA	TGGTCCAGGGTTTCTTACTCC
Mouse α-SMA	TGGCTATTCAGGCTGTGCTGTC	CAATCTCACGCTCGGCAGTAGT
Mouse Col1α1	GAGCGGAGAGTACTGGATCG	GCTTCTTTTCCTTGGGGTTC
Mouse PPARɣ	AGCCCTTTACCACAGTTGATTTCTCC	GCAGGTTCTACTTTGATCGCACTTTG
Rat GAPDH	TGTCACCAACTGGGACGATA	GGGGTGTTGAAGGTCTCAAA
Rat α-SMA	GCGTGGCTATTCCTTCGTGACTAC	CATCAGGCAGTTCGTAGCTCTTCTC
Rat Col1α1	TGTTGGTCCTGCTGGCAAGAATG	GTCACCTTGTTCGCCTGTCTCAC
Human GAPDH	CAGGAGGCATTGCTGATGAT	GAAGGCTGGGGCTCATTT
Human α-SMA	TCCGGAGCGAAATACTCTG	CCCGGCTTCATCGTATTCCT
Human Col1α1	CCACCAATCACCTGCGTACA	CACGTCATCGCACAACACCT
Mouse Nedd4	CCGAGTATAGTGGTCAGGCTGTC	TGGCTGGTGGCTGGATTTCC

**Table 2:** Antibodies.

Antibody	Manufacturer
GAPDH	Proteintech, Wuhan, China
α-SMA	Proteintech, Wuhan, China
Col1α1	Abcam, Cambridge, UK
PPARγ	Abcam, Cambridge, UK
Smad3	Abcam, Cambridge, UK
p-Smad3	Cell Signaling Technology, Danvers, MA, USA
Nedd4	Proteintech, Wuhan, China
Lamin B1	Proteintech, Wuhan, China
Ubiquitin	Proteintech, Wuhan, China

## Data Availability

The datasets used and analyzed during the current study are available from the corresponding authors on reasonable request.

## Abbreviations

The following abbreviations are used in this manuscript:

IPF	Idiopathic pulmonary fibrosis
TGF-β	Transforming growth factor-β
PPARγ	Peroxisome proliferator-activated receptor gamma
BLM	Bleomycin
HE	Hematoxylin and eosin staining
RT-qPCR	Real-time quantitative reverse transcription polymerase chain reaction
WB	Western blotting
HFL1	Human fetal lung fibroblasts
CCK-8	Cell Counting Kit-8
Co-IP	Co-immunoprecipitation
ECM	Extracellular matrix
p-Smad3	Phosphorylated Smad3
α-SMA	Alpha smooth muscle actin
